# Expression of ferritin-like protein in *Listeria monocytogenes* after cold and freezing stress

**DOI:** 10.1007/s12223-012-0172-z

**Published:** 2012-06-07

**Authors:** Hanene Miladi, Abdelaziz Soukri, Amina Bakhrouf, Emna Ammar

**Affiliations:** 1 Laboratoire d’Analyses, Traitement et Valorisation des Polluants de l’Environnement et des Produits, Faculté de Pharmacie, rue Avicenne, 5000 Monastir, Tunisia; 2 Laboratoire de Physiologie et de Génétique Moléculaire (PGM), Faculté des Sciences, Ain-Chock, , BP 5366 Maarif Casablanca, Morocco; 3 Unité de Recherche Gestion des Environnements Urbains et Côtiers–LARSEN, Ecole Nationale d’Ingénieurs de Sfax (Tunisie), B.P. «W», 3038 Sfax, Tunisia

## Abstract

The cold shock protein family consists of the transfer of the foodborne pathogen *Listeria monocytogenes* from 37 to 4 and −20 °C and was characterized by the sharp induction of a low molecular mass protein. This major cold shock protein ferritin-like protein (Flp) has an important role in regulation of various microbial physiological processes. Flp have a molecular mass of about 18 kDa, as observed on SDS–PAGE. The purification procedure including ammonium sulfate fractionation was used. Monospecific polyclonal antibodies raised in rabbits against the purified new Flp immunostained a single 18-kDa Flp band in extracts from different cytoplasmic proteins blotted onto nitrocellulose. A 411-bp cDNA fragment that corresponds to an internal region of an *flp* gene was obtained by RT-PCR. Our result indicated a surexpression of major cold shock protein and an important increase in flp mRNA amount after a downshift temperature especially at −20 °C.

## Introduction

The gram-positive foodborne bacterial pathogen *Listeria monocytogenes* is a significant public health and food safety problem worldwide. Infection of pregnant women, infants, old people, and individuals of immunocompromised status with this bacterium can lead to listeriosis, a disease condition that can induce severe illnesses and relatively high mortality rates (Arguedas-Villa et al. 2010; Posfay-Barbe and Wald 2009; Swaminathan and Gerner-Smidt 2007). The control of this bacterium during production and storage of processed food products is one of the critical measures in public protection against *L. monocytogenes* infection. This task however remains tough due to environmental ubiquity as well as natural resistance of this bacterium to environmental stress and various microbial control measures applied in food production (Arguedas-Villa et al. 2010; Gandhi and Chikindas 2007).

The use of refrigeration in food processing and conservation for extending the shelf life of foodstuff results in the enrichment of the contamination flora in psychrotrophic microorganisms. *L. monocytogenes* is considered as a psychrotolerant bacterium because of its ability to grow at temperatures as low as 0.5 to 3 °C depending on the strains (Junttila et al. 1988; Walker et al. 1990). The adaptation of microorganisms to downshifts in temperature involves changes in the synthesis of some cellular proteins and in the structural organization of the cell wall.

Some studies describe the cold shock response of *L. monocytogenes*, but to date, no cold shock protein (CSP) has been identified in this pathogen. Phan-Thanh and Gormon (1995) reported that the analysis of two-dimensional gel electrophoresis (2-DE) protein patterns in the 2 h following a cold shock from 25 to 4 °C revealed the overexpression of 38 polypeptides, whereas Bayles et al. (1996) by the same method detected 12 CSPs after a 37 to 5 °C downshift. Surprisingly, Phan-Thanh and Gormon (1997) noted that six of the *L. monocytogenes* CSPs were also overexpressed by a heat shock from 25 to 49 °C. Furthermore, it has been suggested that CSPs play a role in protection against freezing (Wouters et al. 1999). CSPs are not only induced at low temperature but also by other environmental stresses. CSPA, the major CSP in *Escherichia coli*, was found to be expressed when the organism left the stationary phase (Brandi et al. 1999).

The CSP family consists of small, highly conserved, and structurally related nucleic acid-binding proteins that presumably have important roles in regulation of various microbial physiological processes (Schmid et al. 2009; Ermolenko and Makhatadze 2002). These proteins are widely distributed among prokaryotes, including *L. monocytogenes*, and are often encoded through differentially regulated multiple gene families per organism (Phadtare 2004; Wang et al. 1999; Graumann and Marahiel 1998). CSPs are thought to serve as nucleic acid chaperones that bind RNA and DNA and thus may facilitate the control of processes such as replication, transcription, and translation within bacterial cells (Phadtare et al. 1999).

In this study, we report results on the strong overexpression of an 18-kDa protein after a 37 to 4 °C and to −20 °C cold shock. The isolation and characterization of the ferritin-like protein (Flp) from *L. monocytogenes* and Western blot analyses using monospecific polyclonal antibodies against this Flp protein. The Flp is recognized by an antiserum against this protein in the different conditions of cold and freezing stress. Finally, the expression of the gene encoding this protein has been investigated by semiquantitative RT-PCR before and after cold and freezing stress.

## Material and methods

### Bacterial strain isolation and biochemical characterization

Two *L. monocytogenes* strains isolated from meat according to the French standard (NF V 08–055) (Miladi et al. 2008) and *L. monocytogenes* ATCC 19115 were used in this study. The cells were preserved on 20 % glycerol at −80 °C and cultivated on tryptic soy agar—yeast extract (TSA-YE; Bio-Rad) at 37 °C prior to use. The growth of each strain was initiated from a single colony applied to 10 mL of TSA-YE broth cultures and incubated for 16 h at 37 °C in a shaking incubator (220 rpm). This step gave stationary growth stage cultures equivalent to approximately 10^9^ CFU/mL in three strains.

### Cold stress exposure and sample collection

Stationary 75-mL TSA-YE phase cultures comprising *L. monocytogenes* ATCC 19115, strain 1 and strain 2, respectively, were prepared by incubation for 16 h at 37 °C in a shaking incubator as described. Cultures prepared in this way were then centrifuged (5 min at 4,000×*g*), and the *Listeria* cell pellets were resuspended in 75 mL of fresh TSA-YE, which was subdivided into 5-mL aliquots that were incubated at 37 °C (control samples), at 4 °C (cold stress) and at −20 °C (freezing stress) for 1, 2, 3, 4, and 24 h. After stress, 1.5-mL sample aliquots were centrifuged (5 min at 4,000×*g*) at 4 °C (for cold and freezing stress) or room temperature (for control samples).

### Protein purification

The Flp was purified to electrophoretic homogeneity from crude cell extracts by the procedure previously described (Soukri et al. 1995, 1996). All steps were performed at 4 °C. Centrifugations were carried out at 13,000×*g* for 45 min.

### Preparation of crude extracts

After cold and freezing stress, cells were recovered by centrifugation (13,000×*g*, 5 min), and the pellets were suspended in 50 μL of extraction buffer (10 mmol/L Tris HCl, pH 6.8, 0.1 mmol/L EDTA, 5 mmol/L MgCl2) supplemented with 2 mg/mL lysozyme. Samples were incubated 30 min at 25 °C and stored at −20 °C (Hebraud and Guzzo 2000).

### Ammonium sulfate fractionation

Cell-free extracts from *L. monocytogenes* were obtained as described before. A two-step ammonium sulfate precipitation was applied to the cell-free extract in order to recover the 40–60 % saturation fraction. After a 2-h dialysis in a microdialyzer apparatus against a continuous flow of the extraction buffer and the fractions containing the Flp protein are ready for the identification (Hebraud and Guzzo 2000).

### Polyacrylamide gel electrophoresis

Sodium dodecyl sulfate–polyacrylamide gel electrophoresis (SDS–PAGE) was performed as described by Laemmli (1970) on one-dimensional 12 % polyacrylamide slab gels containing 0.1 % SDS. Gels were run on a miniature vertical slab gel unit (Hoefer Scientific Instruments). After electrophoresis, gels were stained with Coomassie Brilliant Blue R-250 at 0.2 % (*w*/*v*) in the mixture of methanol/acetic acid/water (4:1:5, *v*/*v*/*v*) for 30 min at room temperature. The apparent subunit molecular weight was determined by measuring relative mobilities and comparing with the following prestained SDS–PAGE molecular weight standards (Low Range MW, Bio-Rad): myosin, 212 kDa; β-galactosidase, 116 kDa; phosphorylase B, 97.4 kDa, BSA, 66 kDa; ovalbumin, 45 kDa; carbonic anhydrase, 29 kDa; trypsin inhibitor, 20 kDa; and lysozyme, 14.4 kDa.

### Preparation of polyclonal antiserum

Iberian ribbed newts, *Pleurodeles waltl* (Amphibia, Batrachia, Caudata wUrodelax, Salamandridae), are originated from Morocco. The animals used in this study were from our breeding stocks.

Polyclonal antibodies were raised in New Zealand White rabbits to glyceraldehyde-3-phosphate dehydrogenase (GAPDH) that had been purified to electrophoretic homogeneity from *Pleurodeles* skeletal muscle. The purified enzyme (approximately. 0.3 mg) was mixed with Freund’s complete adjuvant and injected subcutaneously to rabbits in multiple places as described by Vaitukaitis (1981). Rabbits were boosted four times at 3-week intervals, and bleeding was done 10 days after.

### Western blot analysis

Proteins were separated by SDS–PAGE as described previously. Separated protein bands were electrophoretically transferred from the gel slab to a nitrocellulose membrane (Schleicher & Schuell) using a Bio-Rad Trans-Blot system. Transferred proteins were then visualized by prestaining in 0.2 % (*w*/*v*) Ponceau Red in trichloroacetic acid. The nitrocellulose membrane was then incubated for 1 h in blocking solution containing 5 % (*w*/*v*) nonfat dry milk, 50 mmol/L Tris–HCl (pH 7.5), 150 mmol/L NaCl, 0.01 % (*w*/*v*) NaN3, and 0.05 % (*v*/*v*) Tween-20, followed by incubation with the anti-GAPDH antiserum (1:1,000 dilution) as the first antibody. Western blots were eventually visualized by coupled immunoreaction with peroxidase-conjugated goat antirabbit IgG (Sigma Chemical Co.) as the second antibody using 3,3′-diaminobenzidine as the chromogenic substrate.

### Nucleic acid techniques

#### RNA isolation and reverse transcriptase polymerase chain reaction

Total RNA was isolated from control and stressed cells by SV total RNA isolation system (Promega, France) according to the manufacturer’s instructions. First-strand cDNA was produced by reverse transcription (RT) using murine Moloney leukemia virus reverse transcriptase (Invitrogen) in conjunction with 100 ng total RNA and the reverse primer named Flp R; 5′-TTTGAACATCCAGATGTGTT-3′ for 45 min at 42 °C. An aliquot from this template (1/10 of the reaction) was used in a subsequent polymerase chain reaction (PCR) using Taq DNA polymerase (Promega), Flp R, and forward primer named Flp F; 5′-GAGTTCCTGAATCACCAGGT-3′. The PCR conditions of the *flp* gene included an initial step (94 °C for 5 min), followed by 30 cycles of denaturation (94 °C for 1 min), annealing (56 °C for 1 min) and extension (72 °C for 1 min), and concluded at the end of cycling by a final extension (72 °C for 10 min).

PCR products (7 μL) were analyzed on 1 % agarose gel stained with ethidium bromide (0.5 mg/mL) at 100 V for 45 min and viewed under ultraviolet transillumination. The amplification products were photographed and their sizes determined with a 100-bp molecular size marker (Promega). Quantitative analysis of DNA bands was performed using imaging software (Gene Tools, Sygene, UK).

#### Statistical analysis

Statistical analysis was performed using the SPSS 13.0 statistics package for Windows. The differences in the level of *flp* gene expression were examined by the Friedman test, followed by the Wilcoxon signed ranks test. *P* values of <0.05 were considered significant.

## Results and discussion

### Identification of the major protein induced by cold shock

**Fig. 1:**
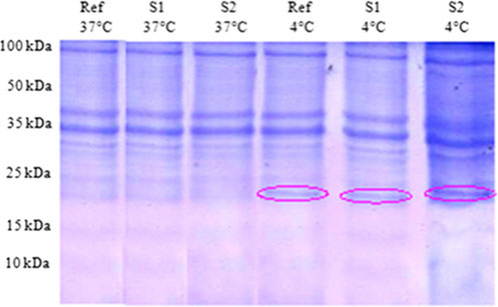
The protein profile of three strains before and after induction of cold stress at 4 °C by SDS–PAGE 12 %

After the temperature downshift, we showed that protein synthesis was affected by the cold shock at 4 °C. We noted an overexpression of proteins visualized on SDS–PAGE. We notice a difference in the protein profile after cold shock at 4 °C in the gel (Fig. 1).

**Fig. 2:**
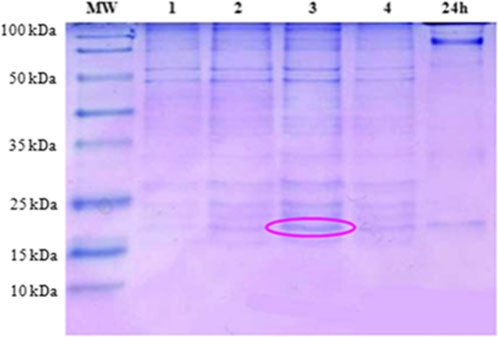
The protein profile of *L. monocytogenes* ATCC 19115 after cold shock at −20 °C during 1, 2, 3, 4, and 24 h by SDS–PAGE 12 %

**Fig. 3:**
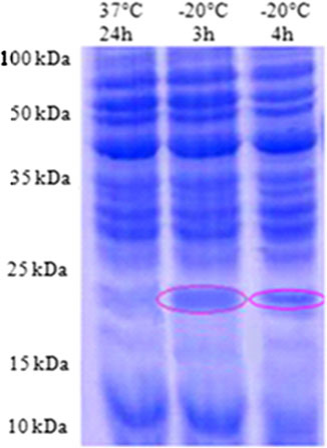
The protein profile of S1 after cold shock at −20 °C by SDS–PAGE 12 %

**Fig. 4:**
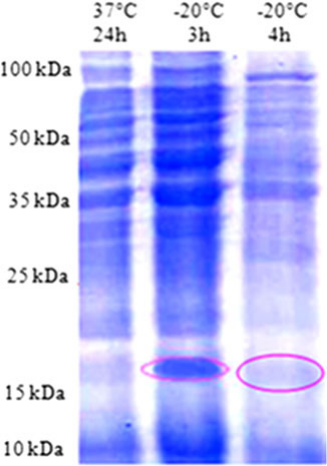
The protein profile of S2 after cold shock at −20 °C by SDS–PAGE 12 %

Freezing stress also strongly affected the profile of protein in *L. monocytogenes*. In different strains used in this study, *L. monocytogenes* ATCC 19115 and two isolated strains (S1 and S2), the 18-kDa CSP was particularly overexpressed several hours after the downshift at −20 °C. Figures 2, 3, and 4 indicate the surexpression of a very visible strip after induction of stress by the cold shock at −20 °C during 3 h, of included size between 15 and 25 kDa, and one estimates that it is of 18 kDa. According to the bibliography, it is the cold shock protein such as the ferritin-like protein. Such an observation led to qualify this small protein as cold acclimation protein rather than CSPs (Hébraud and Potier 1999; Hebraud and Guzzo 2000). However, it is obvious that this small protein could play an important role in the response to numerous stresses, which means that the gene could be submitted to a regulatory system with multiple signals. The analysis of autoradiograms from SDS–PAGE of cold shock revealed the induction of many proteins but is more pronounced and 18 kDa (Fig. 1). Most of these CSPs presented similar molecular masses with the 38 or 12 CSPs previously identified by 2-DE from the strains *L. monocytogenes* EGD (Phan-Thanh and Gormon 1995) or *L. monocytogenes* 10403S (Bayles et al. 1996), respectively. Among the low molecular mass proteins, a CSP close to 18 kDa was noticed in the three different strains of *L. monocytogenes* (17.6 to 19 kDa) as well as in *Listeria innocua* CHUT 861156 (Phan-Thanh and Gormon 1995) and always presented the highest level of induction. Concomitantly, Phan-Thanh and Gormon (1995) showed a very strong induction of this *L. monocytogenes* EGD small protein (more than 50-fold) by a heat shock treatment from 25 to 49 °C and also a more or less important induction by different stressing agents, including SDS (2.2-fold), deoxycholate (14.5-fold), and ethanol (2.2-fold) (Phan-Thanh and Gormon 1997). In *L. monocytogenes* LO28, the 18-kDa CSP was particularly overexpressed several hours after the downshift at 5 °C.

In bacteria cold shocked at −20 °C, Flp could be detected, similarly in cultures grown at either 4 or 37 °C, suggesting that Flp is subjected to multiple forms of regulation. This phenomenon was also described in the study of Hebraud and Guzzo (2000) who found that transcription of the ferritin gene was barely detectable at 30 °C but was induced in cells that were either heat- or cold shocked at 49 and 5 °C, respectively (Hebraud and Guzzo 2000). Similarly, in a study on adaptive changes to high and low temperatures in *Listeria*, Phan-Thanh and Gormon (1995) described a 17.6-kDa polypeptide that was induced under conditions of both cold and heat shock (Phan-Thanh and Gormon 1995).

### Identification and purification of the major CSP

**Fig. 5:**
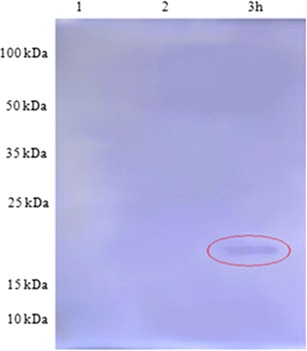
Immunodetection by Western blot of the Flp protein of the reference strain after transfer on membrane of nitrocellulose: *1* Ref at 37 °C, *2* Ref at 4 °C, *3* Ref at −20 °C

In order to characterize the *L. monocytogenes* Flp, we have undertaken its purification. Cytoplasmic proteins were subjected to a two-step ammonium sulfate precipitation. The 40–60 % saturation fraction allowed eliminating 52 % of total cellular proteins while recovering entirely the Flp as assessed by SDS–PAGE. We have produced rabbit polyclonal antibodies using purified *L. monocytogenes* Flp protein. These antibodies selectively reacted by the immunoblotting procedure with a single immunoreactive band in purified preparations. Figure 2 shows that the relative molecular mass of the detected protein (18 kDa) is the expected one for the Flp monomer. The same protein band was recognized by the anti-Flp antiserum in fraction of protein obtained after 3 h at freezing stress (−20 °C; Fig. 5). No protein bands were detected by immune rabbit serum with protein gotten after culture of 37 or 4 °C. This result is in agreement with those observed on SDS–PAGE.

In bacteria cold shocked at −20 °C, Flp could be detected, similarly in cultures grown at either 4 or 37 °C, suggesting that Flp is subjected to multiple forms of regulation. This phenomenon was also described in the study of Hebraud and Guzzo (2000) who found that transcription of the ferritin gene was barely detectable at 30 °C but was induced in cells that were either heat- or cold shocked at 49 and 5 °C, respectively (Hebraud and Guzzo 2000). Similarly, in a study on adaptive changes to high and low temperatures in *Listeria*, Phan-Thanh and Gormon described a 17.6-kDa polypeptide that was induced under conditions of both cold and heat shock (Phan-Thanh and Gormon 1995).

The analysis of autoradiograms from SDS–PAGE of cold shock revealed the induction of many proteins but is more pronounced for 18 kDa (Fig. 1). Most of these CSPs presented similar molecular masses with the 38 or 12 CSPs previously identified by 2-DE from the strains *L. monocytogenes* EGD (Phan-Thanh and Gormon 1995) or *L. monocytogenes* 10403S (Bayles et al. 1996), respectively. Among the low molecular mass proteins, a CSP close to 18 kDa was noticed in the three different strains of *L. monocytogenes* (17.6 to 19 kDa) as well as in *L. innocua* CHUT 861156 (Phan-Thanh and Gormon 1995) and always presented the highest level of induction. Concomitantly, Phan-Thanh and Gormon (1995) showed a very strong induction of this *L. monocytogenes* EGD small protein (more than 50-fold) by a heat shock treatment from 25 to 49 °C and also a more or less important induction by different stressing agents, including SDS (2.2-fold), deoxycholate (14.5-fold), and ethanol (2.2-fold) (Phan-Thanh and Gormon 1997). In *L. monocytogenes* LO28, the 18-kDa CSP was particularly overexpressed several hours after the downshift at 5 °C.

### Effect of cold shock on Flp mRNA abundance

**Fig. 6:**
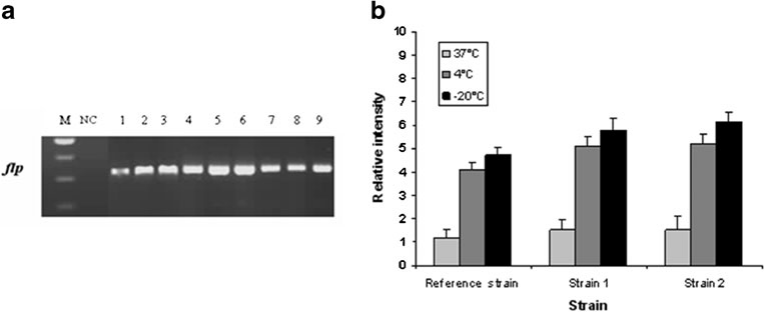
Agarose gel analysis (1 %) of *flp* expression of *L. monocytogenes* before and after cold and freezing stress. **a** *M* 100-bp DNA ladder (Promega), *NC* negative control, *1* Ref_37_, *2* Ref_4_, *3* Ref_−20_, *4* S1_37_, *5* S1_4_, *6* S1_−20_, *7* S2_37_, *8* S2_4_, *9* S2_−20_. **b** The relative expression of *flp* gene

Ferritin-like protein from *L. monocytogenes* is believed to act as a cold shock protein (Bozzi et al. 1997; Hebraud and Guzzo 2000). After cold and freezing stress, we observed an increase in the expression level of *flp* gene in *L. monocytogenes*, and we noted that the expression level of this gene at −20 °C increased more than those at 4 and 37 °C in tested stains. However, after freezing stress, the relative intensity is the highest and increased by 3.512 ± 0.354, 4.221 ± 0.431, and 4.603 ± 0.533 for reference strain, S1, and S2 respectively (Fig. 6) compared then at 37 °C. On the other hand, we noted that the expression of *flp* gene at 4 °C was more important than at 37 °C. Statistical analysis revealed a significant difference between the level of expression at 37, 4, and −20 °C (*P* < 0.05). Flp mRNA was detected at a significant level in the absence of stress, but after cold shock, a sharp increase of Flp mRNA level was observed. Moreover, the abundance of the specific mRNA also increased significantly after heat shock, suggesting that Flp could be a heat shock protein. These results strongly suggest that the *L. monocytogenes* LO28 Flp corresponds to the 19-kDa multistress protein pointed out by Phan-Thanh and Gormon (1995, 1997).

## Conclusion

Listerial ferritin-like protein levels were strongly dependent on growth phase. A complex pattern of Flp expression was observed in cultures growing at −20, 4, and 37 °C. In bacteria grown at 37 °C, Flp could be detected, but was readily detectable in cultures at either 4 or −20 °C, suggesting that Flp is subjected to multiple forms of regulation. It is also noteworthy that very different organisms can present cold-induced Flp such as the 20.2-kDa Flp of the cyanobacterium *Anabaena variabilis* (Sato 1991). In *L. monocytogenes*, the Flp seems to act as a multistress protein, the induction of which occurs in response to very different stressing agents. Regulation of Flp synthesis may occur at the transcriptional level considering the increase of *flp* mRNA amount upon heat and cold shock. Bozzi et al. (1997) reported that *L. innocua* ferritin was able to oxidize and sequester about 500 iron atoms. So, it can be assumed that various stressing conditions impaired the iron repository or oxidation in cells, and an elevated level of ferritin allows a metabolic compensation to these phenomena. Obviously, more investigations are necessary to understand the involvement of ferritin in response to various stressing conditions.

## References

[CR1] Arguedas-Villa C, Stephan R, Tasara T (2010). Evaluation of cold growth and related gene transcription responses associated with *Listeria monocytogenes* strains of different origins. Food Microbiol.

[CR2] Bayles DO, Annous BA, Wilkinso BJ (1996). Cold stress induced in *Listeria monocytogenes* in response to temperature down shock and growth at low temperatures. Appl Environ Microbiol.

[CR3] Bozzi M, Mignogna G, Stefanini S, Barra D, Longhi C, Valenti P, Chiancone E (1997). A novel non-heme iron-binding ferritin related to the DNA-binding proteins of the Dps family in *Listeria innocua*. J Biol Chem.

[CR4] Brandi A, Spurio R, Gualerzi CO, Pon CL (1999). Massive presence of the *Escherichia coli* ‘major cold-shock protein’ CspA under non-stress conditions. EMBO J.

[CR5] Ermolenko DN, Makhatadze GI (2002). Bacterial cold-shock proteins. Cell Mol Life Sci.

[CR6] Gandhi M, Chikindas ML (2007). *Listeria*: a foodborne pathogen that knows how to survive. Int J Food Microbiol.

[CR7] Graumann PL, Marahiel MA (1998). A superfamily of proteins that contain the cold-shock domain. Trends Biochem Sci.

[CR8] Hebraud M, Guzzo J (2000). The main cold shock protein of *Listeria monocytogenes* belongs to the family of ferritin-like proteins. FEMS Microbiol Lett.

[CR9] Hébraud M, Potier P (1999) Cold shock response and low temperature adaptation in psychrotrophic bacteria. J Mol Microbiol Biotechnol 1(2):211–219 10943552

[CR10] Junttila JR, Niemalä SI, Hirn J (1988). Minimum growth temperature of *Listeria monocytogenes* and non-haemolytic *Listeria*. J Appl Bacteriol.

[CR11] Laemmli IK (1970). Cleavage of structural proteins during the assembly of the head of bacteriophage T4. Nature.

[CR12] Miladi H, Chaieb K, Bakhrouf A, Elmnasser N, Ammar E (2008). Freezing effects on survival of *Listeria monocytogenes* in artificially contaminated cold fresh-salmon. Ann Microbiol.

[CR13] Phadtare S (2004). Recent developments in bacterial cold-shock response. Curr Issues Mol Biol.

[CR14] Phadtare S, Alsina J, Inouye M (1999). Cold-shock response and cold-shock proteins. Curr Opin Microbiol.

[CR15] Phan-Thanh L, Gormon T (1995). Analysis of heat and cold shock proteins in *Listeria* by two-dimensional electrophoresis. Electrophoresis.

[CR16] Phan-Thanh L, Gormon T (1997). Stress proteins in *Listeria monocytogenes*. Electrophoresis.

[CR17] Posfay-Barbe KM, Wald RW (2009). Listeriosis. Semin Fetal Neonatal Med.

[CR18] Sato N (1991) Hypothetical 20.2-kDa low temperature-induced protein. EMBL/GenBank/DDBJ accession number D01016

[CR19] Schmid B, Klumpp J, Raimann E, Loessner MJ, Stephan R, Tasaral T (2010). Role of cold shock proteins in growth of *Listeria monocytogenes* under cold and osmotic stress conditions. Appl Env Microbiol.

[CR20] Soukri A, Valverde F, Hafid N, Elkebbaj MS, Serrano A (1995). Characterization of muscle glyceraldehyde-3- phosphate dehydrogenase isoforms from euthermic and induced hibernating *Jaculus orientalis*. Biochim Biophys Acta.

[CR21] Soukri A, Hafid N, Valverde F, ElKebbaj MS, Serrano A (1996). Evidence for a posttranslational covalent modification of liver glyceraldehyde-3-phosphate dehydrogenase in hibernating jerboa (*Jaculus orientalis*). Biochim Biophys Acta.

[CR22] Swaminathan B, Gerner-Smidt P (2007). The epidemiology of human listeriosis. Microbes Infect.

[CR23] Vaitukaitis JL (1981) Production of antisera with small doses of immunogen: multiple intradermal injections. Methods Enzymol 73:46–52 10.1016/0076-6879(81)73055-6 7300686

[CR24] Wang N, Yamanaka K, Inouye M (1999). CspI, the ninth member of the CspA family of *Escherichia coli*, is induced upon cold shock. J Bacteriol.

[CR25] Walker SJ, Archer P, Banks JG (1990). Growth of *Listeria monocytogenes* at refrigeration temperatures. J Appl Bacteriol.

[CR26] Wouters JA, Jeynov B, Rombouts FM, de Vos WM, Kuipers OP, Abee T (1999). Analysis of the role of 7 kDa cold-shock proteins of *Lactococcus lactis* MG1363 in cryoprotection. Microbiol.

